# Strengthening Families, Empowering TGD Lives through Inclusive Mental Health Care

**DOI:** 10.1192/j.eurpsy.2026.11722

**Published:** 2026-07-31

**Authors:** J. Hubler, A. Jambrosic Sakoman, A. Vuk, I. Barun, L. Mijalkovic, M. Supe, J. Pravdic, V. V. Dogas

**Affiliations:** 1 Addiction Day Hospital, University Psychiatric Hospital Sveti Ivan; 2 Department of Psychotic Disorders, University Psychiatric Hospital Vrapce; 3 Department of integrativne psychiatry; 4 Eating Disorders Day Hospital; 5 Department of Psychotherapeutic Treatment, University Psychiatric Hospital Sveti Ivan, Zagreb, Croatia

## Abstract

**Introduction:**

Throughout their lives, transgender and gender-diverse (TGD) people are exposed to multiple challenges such as human rights violations, restricted access to healthcare, hostile narratives in society, and rejection from their families. Such continuous exposure to stigma, discrimination, and violence increases minority stress, negatively affecting both mental and physical health (Frost & Meyer, 2023). When family support is absent, the risks intensify, leading to higher rates of depression, anxiety, self-harm, and suicidality (Malpas, Pellicane & Glaeser, 2022). Research and clinical practice with families of TGD youth confirm that family acceptance and support are key to psychosocial wellbeing, mental health, and quality of life (McGregor et al., 2024; Başar et al., 2016; Campbell et al., 2024). Family therapy is standard practice in countries where gender-affirming healthcare is available, and the WPATH Standards of Care (Version 8) recommend involving parents in processes that strengthen children’s psychological safety and support.

**Objectives:**

The aim is to introduce different modalities of therapeutic work with families of TGD people, including counseling, family therapy, and multi-family therapy. These interventions are provided both within the public healthcare system through the BOJE Mental Health Support Programme for LGBTQIA+ people at the University Psychiatric Hospital Sveti Ivan, and in collaboration with civil society organizations.

**Methods:**

Building on insights from previous research, the Mental Health Support Programme BOJE (Colours) was launched to offer comprehensive support tailored to the needs of LGBTQIA+ individuals within the Croatian public mental healthcare system. The programme consists of a counseling center, an outpatient clinic, a three-month structured group cycle (combining psychoeducation, group psychotherapy, and art therapy), ongoing support groups, and psychotherapeutic work with families, implemented in partnership with civil society organizations.

**Results:**

In family therapy, working with a transgender member opens a safe space for constructive dialogue, identity exploration, and adapting to shifts in family dynamics. Our expirience shows that these interventions lead to improved communication, greater openness, and reduced feelings of isolation.

**Conclusions:**

The BOJE programme demonstrates how structured interventions can buffer the impact of minority stress. In particular, therapeutic work with families highlights the critical role of acceptance in promoting wellbeing. Our experience shows that these interventions nurture mutual understanding, acceptance, and hope — demonstrating that positive change is possible and support attainable.

**Disclosure of Interest:**

None Declared

